# Supplementary material for: An Allee-based distributed algorithm for microbial whole-cell sensors
Source: NPJ Syst Biol Appl. 2024 Apr 22;10:43. doi: 10.1038/s41540-024-00363-3 (PMC11035582; doi:10.1038/s41540-024-00363-3)
Supplement: Supplementary file 1 — Supplementary Material [file 41540_2024_363_MOESM1_ESM.pdf]

Supplementary Material

Mathematical Proofs

Proof of Theorem 1

We start this section with a recall of the model parameters of reactions (Set), (Hold), and (Reset). Let the rare event detection rate σ be in $\mathbb{R}_+$, the reset rate constant ρ be in $\mathbb{R}_{>0}$, and the hold rate parameter κ be in $\mathbb{R}_{>0}$. Let the maximum population density P be in $\mathbb{R}_{>0}$. Denoting by $H \in \mathbb{R}_+$ the density of H cells, its behavior over time $t \in \mathbb{R}_+$ is given by

$$\frac{dH}{dt} = \sigma \cdot (P - H) + \kappa \frac{H^n}{H^n + K^n}(P - H) - \rho H \quad . \tag{1}$$

We refer to the right-hand side of Equation (1) as the function s_σ from $\mathbb{R}_+$ to $\mathbb{R}$, emphasizing the fact that it depends on σ. In particular, $s_\sigma = s_0 + \sigma \cdot (P - H)$.

The equilibrium points of $H(t)$ are the roots of s_σ. We start the analysis of the roots by establishing conditions for when s_σ has a unique local minimum and a unique local maximum. Those will be later on used to establish the requirements on s_σ to have two stable equilibrium points. We begin with a technical lemma:

Lemma 1. *Let $\Pi(H) = C - aH - bH^n + cH^{n+1}$ with $H \geq 0$, $C > 0$, $a > 0$, $b > 0$, $c > 0$, and $n > 0$. For $H \geq 0$, one of the following is true:*

1. *There exists an interval $I =]H_{inc}, H_{dec}[$, such that $\Pi(H) < 0$ if and only if H is in I. Furthermore, one has $\Pi(H) = 0$ if and only if $H = H_{inc}$ or $H = H_{dec}$.*

2. *The polynomial Π is positive with the exception of at most one zero in $[0, +\infty[$.*

Proof. It is $\frac{d\Pi}{dH} = -a - nbH^{n-1} + (n+1)cH^n$. Further, if $H > 0$,

$$\frac{d\Pi}{dH} < 0 \Leftrightarrow a + nbH^{n-1} > (n+1)cH^n \Leftrightarrow \frac{a + nbH^{n-1}}{H^n} > (n+1)c \Leftrightarrow \frac{a}{H^n} + \frac{nb}{H} > (n+1)c \tag{2}$$

As $n > 0$, the expression $\frac{a}{H^n} + \frac{nb}{H}$ is strictly decreasing for H within $\mathbb{R}_+$, converges to 0 as H goes to inf, and converges to inf as H goes to 0. Thus, there exists $H_{\mathrm{d3}} > 0$ such that for all H in $]0, H_{\mathrm{d3}}[$, Equation (2) holds, and for all H in $[H_{\mathrm{d3}}, +\infty[$, Equation (2) does not hold. For the remaining value of $H = 0$ that is needed to complete the analysis in the interval $[0, +\infty[$, one has $\frac{d\Pi}{dH}(0) < 0$. It follows that $\frac{d\Pi}{dH}$ is negative in $[0, H_{\mathrm{d3}}[$, positive in $]H_{\mathrm{d3}}, +\infty[$, and zero at H_{d3}. Therefore, Π is strictly decreasing for H within $[0, H_{\mathrm{d3}}]$, strictly increasing for H within $[H_{\mathrm{d3}}, +\infty[$, and there is a unique global minimum for Π at H_{d3}.

Let Q be the following predicate: There exists H_{dr} that is in $[0, H_{\mathrm{d3}}]$ such that $\Pi(H_{\mathrm{dr}}) < 0$. To conclude the proof, one can perform a case distinction over Q and it's negation to show that Q implies statement 1 of the lemma and the negation of Q implies statement 2 of the lemma:

Case Q: Let us assume there exists an $H_{\rm dr}$ that is in $[0, H_{\rm d3}]$ such that $\Pi(H_{\rm dr}) < 0$. As Π is continuous for H in $\mathbb{R}_+$, the intermediate value theorem applies. Thus, as $\Pi(0) = K > 0$, $\Pi(H_{\rm dr}) < 0$, and $\Pi(H_{\rm dr})$ is strictly decreasing for H within $[0, H_{\rm d3}]$, one has that Π has a unique root $H_{\rm dinc}$ in $[0, H_{\rm d3}]$. Also, as $\Pi(H_{\rm dr}) < 0$, $\lim_{H\to+\infty} \Pi(H) = +\infty$, and Π is strictly increasing for H within $[H_{\rm d3}, +\infty]$, one has that Π has another root $H_{\rm ddec}$ in $]H_{\rm d3}, +\infty[$. Thus, there exists an interval $I =]H_{\rm dinc}, H_{\rm ddec}[$, such that $\Pi(H) < 0$ for all $H \in I$ and $\Pi(H) > 0$ for $H \in [0, H_{\rm dinc}[\cup]H_{\rm ddec}, +\infty[$. Let $H_{\rm dinc} = H_{\rm inc}$ and $H_{\rm ddec} = H_{dec}$ for statement 1 to hold.

Case $\neg Q$: Otherwise, there does not exist an $H_{\rm dr}$ in $[0, H_{\rm d3}]$ such that $\Pi(H_{\rm dr}) < 0$. Thus, $\Pi(H_{\rm d3}) \geq 0$. It follows that Π is positive in $[0, +\infty[$ with the exception of at most one zero $H_{\rm d3}$, as Π has a unique global minimum at $H_{\rm d3}$. Thus, statement 2 of the lemma follows.

Since either statement 1 or statement 2 hold, the lemma follows. □

We next provide a definition to distinguish between different types of s_σ:

Definition 1. *We say s_σ is well-shaped if it has a unique local minimum at $H = H_{fmin,\sigma}$ in $\mathbb{R}_{>0}$ and a unique local maximum in $\mathbb{R}_{>0}$ at $H = H_{fmax,\sigma}$, with $H_{fmin,\sigma} < H_{fmax,\sigma}$.*

We also provide the first and second derivatives of s_σ, as they will be used to study the conditions for which s_σ is well-shaped. From the definition of s_σ:

$$s_\sigma(H) = \sigma \cdot (P - H) + \kappa \frac{H^n}{H^n + K^n}(P - H) - \rho H \tag{3}$$

$$\frac{d}{dH} s_\sigma(H) = \kappa \frac{H^{n-1}(nK^nP - nK^nH - K^nH^n - H^{n+1})}{(K^n + H^n)^2} - \sigma - \rho \tag{4}$$

$$\frac{d^2}{dH^2} s_\sigma(H) = \kappa \frac{nK^nH^{n-2}(nPK^n - PK^n - (nK^n + K^n)H - (nP + P)H^n + (n-1)H^{n+1})}{(K^n + H^n)^3} \tag{5}$$

We now prove a lemma that describes the progression of $\frac{ds_\sigma}{dH}$ as H increases.

Lemma 2. *Using the notation from Lemma 1, the derivative $\frac{ds_\sigma}{dH}(H)$ is strictly increasing for H within $[0, H_{inc}]$, strictly decreasing for H within $[H_{inc}, H_{dec}]$, and strictly increasing for H within $[H_{dec}, +\infty[$.*

Proof. From Equation (4), since $nK^nH^{n-2} > 0$ for $H > 0$, the sign of $\Pi(H) = nPK^n - PK^n - (nK^n + K^n)H - (nP + P)H^n + (n-1)H^{n+1}$ in any interval $I_{\rm d}$ determines if $\frac{ds_\sigma}{dH}$ is increasing, decreasing, or neither for H in $I_{\rm d}$.

From Lemma 1 applied to polynomial Π, either the lemma's statement 1 or 2 holds. Assume by means of contradiction that statement 2 holds. Then, $\frac{ds_\sigma}{dH}$ is strictly increasing for H within $[0, +\infty[$. From the definition of s_σ, one has $\frac{ds_\sigma}{dH}(0) = -\sigma - \rho > -\kappa - \sigma - \rho$. However, $\lim_{H\to+\infty} \frac{ds_\sigma}{dH} = -\kappa - \sigma - \rho$. As $\frac{ds_\sigma}{dH}(0)$ is greater than its limit to infinity, one has that $\frac{ds_\sigma}{dH}$ cannot be strictly increasing in H in $[0, +\infty[$; a contradiction to statement 2 of Lemma 1.

Therefore Lemma 1 statement 1 has to hold; concluding the proof. □

We next establish the conditions for s_σ to be well-shaped using the roots of its derivative $\frac{ds_\sigma}{dH}$ in the following lemma:

Lemma 3. *The function s_σ is either:*

1. *Not well-shaped with $\frac{ds_\sigma}{dH}$ being negative in $\mathbb{R}_+$ except for at most one zero.*

2. *Well-shaped with both an interval $I =]H_{d1}, H_{d2}[$, such that for all H in I, one has $\frac{ds_\sigma}{dH}(H) > 0$, and a union of two intervals $S = [0, H_{d1}[\cup]H_{d2}, +\infty[$, such that for all H in S, one has $\frac{ds_\sigma}{dH}(H) < 0$. In this case, $H_{fmin,\sigma} = H_{d1}$ and $H_{fmin,\sigma} = H_{d2}$.*

Proof. From Lemma 2, one has that $\frac{ds_\sigma}{dH}$ is strictly increasing for H within $[0, H_{\text{inc}}]$, strictly decreasing for H within $[H_{\text{inc}}, H_{\text{dec}}]$, and strictly increasing for H within $[H_{\text{dec}}, +\infty[$.

By means of contradiction, let us assume that there exists an H_{db} in $[H_{\text{dec}}, +\infty[$ with $\frac{ds_\sigma}{dH}(H_{db}) \geq 0$. As $\frac{ds_\sigma}{dH}$ is strictly increasing for H within $[H_{\text{dec}}, +\infty[$, if such H_{db} exists in this interval, then for any $H > H_{\text{db}}$, one has $\frac{ds_\sigma}{dH}(H) \geq 0$. However, from the definition of s_σ, one has $\lim_{H\to+\infty} s_\sigma(H) = -\infty$, thus, the existence of such an H_{db} implies a contradiction to the assumption. It follows that for H in $[H_{\text{dec}}, +\infty[$, one has:

$$\frac{ds_\sigma}{dH}(H) < 0 \tag{6}$$

Let T be the following predicate: There exists H_{dp} in $[0, H_{\text{inc}}[$ with $\frac{ds_\sigma}{dH}(H_{\text{dp}}) > 0$. To prove both statements of Lemma 3, one can show that $\neg T$ implies statement 1 to hold while statement 2 not to hold and T implies statement 2 to hold while statement 1 not to hold:

Case $\neg T$: Let us assume that for all H in $[0, H_{\text{inc}}[$, one has $\frac{ds_\sigma}{dH}(H) \leq 0$. Since, from Lemma 2, for all H within $[0, H_{\text{inc}}[$, the derivative $\frac{ds_\sigma}{dH}$ is strictly increasing, and by assumption $\frac{ds_\sigma}{dH}(H) \leq 0$, one has $\frac{ds_\sigma}{dH}(H) < 0$ for any H in $[0, H_{\text{inc}}[$. The inequality becomes a strict one because the interval is open: Assume by means of contradiction that there exists an H_{dint} in $[0, H_{\text{inc}}[$ with $\frac{ds_\sigma}{dH}(H_{\text{dint}}) = 0$, then there also exists an H_{dcont} with $H_{\text{dint}} < H_{\text{dcont}} < H_{\text{inc}}$ for which $\frac{ds_\sigma}{dH}(H_{dcont}) > 0$ by the strict monotonicity of $\frac{ds_\sigma}{dH}$; a contradiction to the initial assumption. Moreover, from Lemma 2, one has that $\frac{ds_\sigma}{dH}$ is strictly decreasing for H within $[H_{\text{inc}}, H_{\text{dec}}]$. Thus, one has that $\frac{ds_\sigma}{dH}(H) < 0$ for H in $]H_{\text{inc}}, H_{\text{dec}}]$. From Equation (6), one has that $\frac{ds_\sigma}{dH}(H) < 0$ for H in $]H_{\text{dec}}, +\infty[$. It follows that $\frac{ds_\sigma}{dH}(H) < 0$ for H in $[0, +\infty[$, except for at most one zero at H_{inc}. Consequently, neither a unique local maximum nor a unique local minimum for s_σ exist in $\mathbb{R}_{>0}$. By the definition of well-shaped, s_σ is not well-shaped.

Case T: Let us assume that there exists an H_{dp} in $[0, H_{\text{inc}}[$ with $\frac{ds_\sigma}{dH}(H_{dp}) > 0$.

Since $\frac{ds_\sigma}{dH}$ is continuous for H in $\mathbb{R}_+$, one can use the intermediate value theorem. From the definition of s_σ and H_{dp}, one has $\frac{ds_\sigma}{dH}(0) < 0$ and $\frac{ds_\sigma}{dH}(H_{\text{dp}}) > 0$. It follows that $\frac{ds_\sigma}{dH}$ has a unique root H_{d1} in $]0, H_{\text{dp}}[\subset [0, H_{\text{inc}}]$.

Further, from Equation (6), for all H in $[H_{\text{dec}}, +\infty[$, one has $\frac{ds_\sigma}{dH}(H) < 0$. Take any H_{dfn} from $[H_{\text{dec}}, +\infty[$, it is $\frac{ds_\sigma}{dH}(H_{\text{dfn}}) < 0$. One can apply the intermediate value theorem to conclude that $\frac{ds_\sigma}{dH}$ has at least one root H_{d2} between H_{dp} with $\frac{ds_\sigma}{dH}(H_{\text{dp}}) > 0$ and H_{dfn} with $\frac{ds_\sigma}{dH}(H_{\text{dfn}}) < 0$. From Lemma 2 we have that $\frac{ds_\sigma}{dH}$ is strictly decreasing in the interval $[H_{\text{inc}}, H_{dec}]$, as well as H_{dp} in $[H_{\text{inc}}, H_{\text{dec}}]$. Also, from Equation (6), for all H in $[H_{\text{dec}}, +\infty[$, it is $\frac{ds_\sigma}{dH}(H) < 0$, and H_{dfn} in $[H_{\text{dec}}, +\infty[$. It follows that the root H_{d2} must be unique.

From Equation (6), one has that $\frac{ds_\sigma}{dH}$ cannot have any roots in $[H_{\text{dec}}, +\infty[$. From the continuity of $\frac{ds_\sigma}{dH}$ in $\mathbb{R}_+$ and the uniqueness of the roots, one has $\frac{ds_\sigma}{dH}(H) > 0$ for any H in $]H_{\text{d1}}, H_{\text{d2}}[$, and $\frac{ds_\sigma}{dH}(H) < 0$ for any H in $[0, H_{\text{d1}}[\cup]H_{\text{d2}}, +\infty[$. Since $\frac{ds_\sigma}{dH}$ only has two roots H_{d1} and H_{d2}, one has that s_σ has a unique local minimum H_{d1} in $\mathbb{R}_{>0}$ and s_σ has a unique local maximum H_{d2} in $\mathbb{R}_{>0}$. One can conclude that s_σ is well-shaped with $H_{\text{fmin},\sigma} = H_{\text{d1}}$ and $H_{\text{fmax},\sigma} = H_{\text{d2}}$.

This concludes the proof. □

We are next studying the roots of s_σ with the goal to use a Lyapunov stability argument for convergence. First, we establish that all roots are in $[0, P[$.

Lemma 4. *The function s_σ does not have any roots greater or equal to P.*

Proof. For all $H \geq P$, one has $-\rho H < 0$, also $\sigma \cdot (P - H) \leq 0$, and $\kappa \frac{H^n}{H^n + K^n}(P - H) \leq 0$. Therefore, $s_\sigma(H) < 0$ and s_σ does not have any roots greater or equal to P. □

Lemma 5. *The local optima of s_σ, for H in $\mathbb{R}_{>0}$, are within $]0, P[$.*

Proof. Since s_σ and $\frac{ds_\sigma}{dH}$ are both continuous and differentiable for H in $\mathbb{R}_+$, a necessary condition for a given H_{lo} to be a local optima in $\mathbb{R}_{>0}$ is $\frac{ds_\sigma}{dH}(H_{\text{lo}}) = 0$. From Equation (4), for any $H \geq P$, one has $\frac{ds_\sigma}{dH}(H) < 0$. Further, from Equation (4), one has $\frac{ds_\sigma}{dH}(0) = -\sigma - \rho$. The lemma's statement follows. □

We will next show in Lemma 6 that s_σ has between one and three roots. In case of three roots, we denote them $H_{\text{ieq},\sigma}$, $H_{\text{ceq},\sigma}$, and $H_{\text{feq},\sigma}$, with $H_{\text{ieq},\sigma} < H_{\text{ceq},\sigma} < H_{\text{feq},\sigma}$. If two roots exist, we denote them $H_{\text{ceq},\sigma}$ and $H_{\text{feq},\sigma}$, with $H_{\text{ceq},\sigma} < H_{\text{feq},\sigma}$. In case of a single root, we denote the root by $H_{\text{feq},\sigma}$. We use σ as an index to emphasize that the roots' values depend on σ. Before showing that there are one to three roots, we state a direct consequence of Lemma 4 and Lemma 5:

Corollary 3. *All of the following roots $H_{ieq,\sigma}$, $H_{ceq,\sigma}$, $H_{feq,\sigma}$, and local optima $H_{fmim,\sigma}$, $H_{fmax,\sigma}$ are in $[0, P[$ whenever they exist.*

We next establish necessary and sufficient conditions for s_σ to have three roots.

Lemma 6. *The following holds:*

- *The function s_σ has three roots if and only if it is well-shaped, $s_\sigma(H_{fmin,\sigma}) < 0$, and $s_\sigma(H_{fmax,\sigma}) > 0$, with $H_{ieq,\sigma} < H_{fmin,\sigma} < H_{ceq,\sigma} < H_{fmax,\sigma} < H_{feq,\sigma}$.*

- *The function s_σ has two roots if and only if it is well-shaped and either $s_\sigma(H_{fmin,\sigma}) = 0$ or $s_\sigma(H_{fmax,\sigma}) = 0$, with $H_{fmin,\sigma} \leq H_{ceq,\sigma} < H_{fmax,\sigma} \leq H_{feq,\sigma}$.*

- *Otherwise, the function s_σ has a single root.*

Proof. One has that s_σ is continuous for H in $\mathbb{R}_+$, so the intermediate value theorem applies. If s_σ is well-shaped, Lemma 3 implies that s_σ is strictly decreasing for H within $[0, H_{\text{fmin},\sigma}]$, strictly increasing for H within $[H_{fmin,\sigma} H_{\text{fmax},\sigma}]$, and strictly decreasing for H within $[H_{fmax,\sigma}, +\infty[$. It follows that s_σ has a

$$\text{unique minimum at } H_{\text{fmin},\sigma} \in [0, H_{\text{fmax},\sigma}] \tag{7}$$

and a

$$\text{unique maximum at } H_{\text{fmax},\sigma} \in [H_{\text{fmin},\sigma}, +\infty[\ . \tag{8}$$

One can distinguish on whether s_σ is not well-shaped and, in case it is well-shaped, the values of s_σ at $H_{\text{fmin},\sigma}$ and $H_{\text{fmax},\sigma}$:

- *If s_σ is not well-shaped*: Lemma 3 statement 1 implies that s_σ is strictly decreasing. Since $s_\sigma(0) = \sigma P$ and $s_\sigma(P) = -\rho P$, s_σ has at least one root in $[0, P]$ due to the intermediate value theorem. The root in $[0, P]$ must be unique because s_σ is strictly decreasing in $[0, \infty[$.

- *If $s_\sigma(H_{fmin,\sigma}) \geq 0$ and $s_\sigma(H_{fmax,\sigma}) < 0$*: this case is a contradiction to the statement that s_σ has a unique minimum at $H_{\text{fmin},\sigma}$ in $[0, H_{\text{fmax},\sigma}]$.

- *If $s_\sigma(H_{fmin,\sigma}) \geq 0$ and $s_\sigma(H_{fmax,\sigma}) \geq 0$*: From (7), only $H_{\text{fmin},\sigma}$ can be a root in $[0, H_{\text{fmax},\sigma}]$. Further, $s(H_{\text{fmax},\sigma}) > s(H_{\text{fmin},\sigma}) > 0$. Since $\lim_{H\to+\infty} s_\sigma(H_{\text{fmax},\sigma}) = -\infty$, and s_σ is strictly decreasing for H within $[H_{\text{fmax},\sigma}, +\infty[$, the function s_σ has only one root in $]H_{\text{fmax},\sigma}, +\infty[$. It follows that s_σ has either one root or, if $s_\sigma(H_{\text{fmin},\sigma}) = 0$, two roots.

- *If $s_\sigma(H_{fmin,\sigma}) < 0$ and $s_\sigma(H_{fmax,\sigma}) \geq 0$*: Since $s_\sigma(0) = \sigma P$, it is $s_\sigma(H_{\text{fmin},\sigma}) < 0$, and s_σ is strictly decreasing for H within $[0, H_{\text{fmin},\sigma}]$, the function s_σ has one root in $[0, H_{\text{fmin},\sigma}[$. From (8), only $H_{\text{fmax},\sigma}$ can be a root in $[H_{\text{fmin},\sigma}, +\infty[$. It follows that s_σ has either one root or, if $s_\sigma(H_{\text{fmax},\sigma}) = 0$, two roots.

- *If $s_\sigma(H_{fmin,\sigma}) < 0$ and $s_\sigma(H_{fmax,\sigma}) > 0$*: From the definition of s_σ, one has $s_\sigma(0) = \sigma P$ and $\lim_{H\to+\infty} s(H) = -\infty$. From the monotocity of the intervals in Lemma 3 statement 2 and the intermediate value theorem, the function s_σ has a root in $[0, H_{\text{fmin},\sigma}[$, another in $]H_{\text{fmin},\sigma} H_{\text{fmax},\sigma}[$, and a last one in $]H_{\text{fmax},\sigma}, +\infty[$. □

As a consequence of Lemma 3 and Lemma 6, we get the following corollaries about the sign of s_σ:

Corollary 4. *If s_σ has three roots, then*

- $s_\sigma(H) > 0$ *within* $[0, H_{ieq,\sigma}[$,
- $s_\sigma(H) < 0$ *within* $]H_{ieq,\sigma}, H_{ceq,\sigma}[$,
- $s_\sigma(H) > 0$ *within* $]H_{ceq,\sigma}, H_{feq,\sigma}[$, *and*
- $s_\sigma(H) < 0$ *within* $]H_{feq,\sigma}, +\infty[$.

Proof. From Lemma 6, it is $H_{\text{ieq},\sigma} < H_{\text{fmin},\sigma} < H_{\text{ceq},\sigma} < H_{\text{fmax},\sigma} < H_{\text{feq},\sigma}$. The corollary then follows from the requirements for $s_\sigma(H_{\text{fmin},\sigma}) < 0$ and $s_\sigma(H_{\text{fmax},\sigma}) > 0$ from Lemma 6, and s_σ being continuous for H in $\mathbb{R}_+$. □

The following lemma establishes how s_σ changes for an increasing parameter σ.

Lemma 7. *For $\varepsilon \in \mathbb{R}_{>0}$, one has $s_{\sigma_1+\varepsilon}(H) > s_{\sigma_1}(H)$ for all $H \in [0, P[$.*

Proof. Let $\sigma_2 = \sigma_1 + \varepsilon$. For any H in $[0, P[$, let $d(H) = s_{\sigma_2}(H) - s_{\sigma_1}(H)$. It is,

$$\begin{aligned} d(H) &= s_{\sigma_2}(H) - s_{\sigma_1}(H) \\ &= \sigma_2 \cdot (P - H) + \kappa \frac{H^n}{H^n + K^n}(P - H) - \rho H - \sigma_1 \cdot (P - H) - \kappa \frac{H^n}{H^n + K^n}(P - H) + \rho H \\ &= (\sigma_2 - \sigma_1)(P - H) \ . \end{aligned}$$

Since $(P - H) > 0$ for $H \in [0, P[$, one has that $d > 0$ if $\sigma_2 > \sigma_1$ from which the lemma follows. □

The next corollary is a direct consequence of Corollary 3 and Lemma 7.

Corollary 5. *Let H be any of $H_{ieq,\sigma}$, $H_{ceq,\sigma}$, $H_{feq,\sigma}$, $H_{fmim,\sigma}$, or $H_{fmax,\sigma}$. For $\varepsilon \in \mathbb{R}_{>0}$, one has $s_{\sigma+\varepsilon}(H) > s_\sigma(H)$.*

In the next two lemmas, we study the impact of σ on the roots.

Lemma 8. *Let s_{σ_1} be a function with three roots. Let $\varepsilon \in \mathbb{R}_{>0}$ and $\sigma_2 = \sigma_1 + \varepsilon$, such that:*

$$\min_{H \in [0, H_{feq,\sigma_1}]} s_{\sigma_1+\varepsilon}(H) < 0$$

Then, s_{σ_2} has three roots as well. Further, for the first two roots, $]H_{ieq,\sigma_2}, H_{ceq,\sigma_2}[\subset]H_{ieq,\sigma_1}, H_{ceq,\sigma_1}[$, and for the third root, $H_{feq,\sigma_1} < H_{feq,\sigma_2}$.

Proof. From Lemma 6, as s_{σ_1} has three roots, it is well-shaped. Thus, the roots H_{ieq,σ_1}, H_{ceq,σ_1}, and H_{feq,σ_1} exist. As $[0, H_{\text{feq},\sigma_1}]$ is a compact set and s_{σ_2} is continuous in this compact set, there exists a value H_{dam} that minimizes s_{σ_2} in the domain $[0, H_{\text{feq},\sigma_1}]$. Furthermore, one has that

$$\min_{H \in [0, H_{\text{feq},\sigma_1}]} s_{\sigma_2}(H) < 0$$

by assumption. Thus

$$s_{\sigma_2}(H_{\mathrm{dam}}) < 0 \ . \tag{9}$$

From Corollary 5 and the fact that $H_{\mathrm{feq},\sigma_1}$ is a root s_{σ_1}, one has that $s_{\sigma_2}(H_{\mathrm{feq},\sigma_1}) > s_{\sigma_1}(H_{\mathrm{feq},\sigma_1}) = 0$. From the definition of s_σ one has $\lim_{H\to+\infty} s_{\sigma_2} = -\infty$. It follows from s_{σ_2} being continuous in $\mathbb{R}_+$ and the intermediate value theorem, that

$$s_{\sigma_2} \text{ has at least one root in }]H_{feq,\sigma_1}, +\infty[\ . \tag{10}$$

Since $s_{\sigma_2}(0) = \sigma_2 P > 0$, $s_{\sigma_2}(H_{\mathrm{dam}}) < 0$ from Equation (9), and $s_{\sigma_2}(H_{\mathrm{feq},\sigma_1}) > 0$, with $0 < H_{\mathrm{dam}} < H_{\mathrm{feq},\sigma_1}$,

$$s_{\sigma_2} \text{ must have at least two roots in } [0, H_{\mathrm{feq},\sigma_1}] \ . \tag{11}$$

It follows that s_{σ_2} has at least three roots. From Lemma 6, s_{σ_2} can have at most three roots. It follows that s_{σ_2} has three roots and s_{σ_2} is also well-shaped, so $H_{\mathrm{ieq},\sigma_2}$, $H_{\mathrm{ceq},\sigma_2}$, $H_{\mathrm{feq},\sigma_2}$, $H_{\mathrm{fmin},\sigma_2}$, and $H_{\mathrm{fmax},\sigma_2}$ exist.

Corollary 4 and Lemma 7 imply that for all H in $[0, H_{\mathrm{ieq},\sigma_1}] \cup [H_{\mathrm{ceq},\sigma_1}, H_{\mathrm{feq},\sigma_1}]$, one has $s_{\sigma_2}(H) > 0$ as $s_{\sigma_1}(H) \geq 0$. From statements (11) and (10), the function s_{σ_2} has two roots in $[0, H_{\mathrm{feq},\sigma_1}]$ as well as one root in $]H_{\mathrm{feq},\sigma_1}, +\infty[$ (in this order). It follows that $]H_{\mathrm{ieq},\sigma_2}, H_{\mathrm{ceq},\sigma_2}[\subset]H_{\mathrm{ieq},\sigma_1}, H_{\mathrm{ceq},\sigma_1}[$ and $H_{\mathrm{feq},\sigma_1} < H_{\mathrm{feq},\sigma_2}$. □

We continue with a definition for the parameters related to the necessary conditions for Theorem 1. We say the parameters are *well-adjusted*, if

$$\max_{H\in[0,P]} \left(\kappa \frac{H^n}{H^n + K^n}(P - H) - \rho H \right) > 0 \ . \tag{12}$$

Noting that s_0 is obtained by setting $\sigma = 0$ in s_σ, one then has

$$s_0 = \kappa \frac{H^n}{H^n + K^n}(P - H) - \rho H \ . \tag{13}$$

We now prove a lemma which will be helpful to show that s_0 has three roots if the parameters are well-adjusted:

Lemma 9. *There exists a non-empty $I =]0, H_{di}[$, such that for every $H \in I$, one has $s_0(H) < 0$.*

Proof. It is $\frac{d}{dH} s_0(H) = \kappa \frac{H^{n-1}(nK^nP - nK^nH - K^nH^n - H^{n+1})}{(K^n+H^n)^2} - \rho$. As $n > 1$, the polynomial $\Pi = H^{n-1}$ has a non-empty interval $I_{\mathrm{d}} =]0, H_{\mathrm{dhn}}[$, such that if H in I_{d}, one has $\Pi(H) < \frac{\rho}{\kappa n K^n P}$. One can then bound $\frac{d}{dH} s_0(H)$ for H in I_{d}:

$$\begin{aligned}
\frac{d}{dH} s_0(H) &< \kappa \frac{\frac{\rho}{\kappa n K^n P}(nK^nP - nK^nH - K^nH^n - H^{n+1})}{(K^n + H^n)^2} \\
&< \kappa \frac{\frac{\rho}{\kappa n K^n P}(nK^nP)}{(K^n + H^n)^2} - \rho \\
&< \left(\frac{1}{(K^n + H^n)^2} - 1 \right) \rho \\
&< 0
\end{aligned}$$

It follows that for $H \in I_{\mathrm{d}}$, $\frac{d}{dH} s_0(H) < 0$. Since $s_0(H) = 0$, it is $s_0(H) < 0$ for H in I_{d}; showing the lemma with $I = I_{\mathrm{d}}$. □

We next prove that s_0 has three roots if the parameters are well-adjusted. This proof allows us to extend the existence of three roots until a critical point using Lemma 8.

Lemma 10. *If the parameters are well-adjusted, the function s_0 has three roots: The smallest one at 0, and two non-zero roots at $\alpha_{di} P$ and $\alpha_{df} P$, with $0 < \alpha_{di} < \alpha_{df} < 1$.*

Proof. It is:

$$s_0 = \kappa \frac{H^n}{H^n + K^n}(P - H) - \rho H \tag{14}$$

As $s_0(0) = 0$, the function s_0 has a root at 0.

From Lemma 9, there is an interval $I =]0, H_{\mathrm{di}}[$, such that for H in I, one has $s_0(H) < 0$. Choosing any H_{dri} in I, one can conclude that

$$s_0(H_{\mathrm{dri}}) < 0 \ . \tag{15}$$

Let $\arg\max_{H \in [0,P]} (\kappa \frac{H^n}{H^n + K^n}(P - H) - \rho H) = H_{\mathrm{dmax}}$. One has that H_{dmax} is well-defined as $[0, P]$ is a compact set and s_0 is continuous in $[0, P]$. From Equation (12), since the parameters are well-adjusted, one has

$$s_0(H_{\mathrm{dmax}}) > 0 \ . \tag{16}$$

Finally, from Lemma 9, since for any H in I, one has $s_0(H) < 0$, one can conclude that

$$H_{\mathrm{dmax}} > H_{\mathrm{dri}} \ , \tag{17}$$

as $s(H_{\mathrm{dmax}}) > 0$ so H_{dmax} must not be in I. From Equations (15), (16), (17), and the intermediate value theorem, the function s_0 must have at least one root in $]H_{\mathrm{dri}}, H_{\mathrm{dmax}}[$.

From the definition of s_0, one has $s_0(P) = -\rho P$, thus

$$s_0(P) < 0 \ . \tag{18}$$

Thus $H_{\mathrm{dmax}} \neq P$ and, together with the definition of H_{dmax},

$$H_{\mathrm{dmax}} < P \ . \tag{19}$$

Thus, from Equations (16), (18), (19), and the intermediate value theorem, the function s_0 must have at least one root in $]H_{\mathrm{dmax}}, P[$.

Since s_0 has at least three roots, Lemma 6 guarantees that there can only be three roots. It follows that, for the roots, $H_{\mathrm{ieq},0} = 0$, $H_{\mathrm{ceq},0}$ in $]H_{\mathrm{dri}}, H_{\mathrm{dmax}}[$, and $H_{\mathrm{feq},0}$ in $]H_{\mathrm{dmax}}, P[$. Setting $\alpha_{\mathrm{di}} = H_{\mathrm{ceq},0}/P$ and $\alpha_{\mathrm{df}} = H_{\mathrm{feq},0}/P$ concludes the proof. □

We will now set $\alpha_{\mathrm{i}} = \alpha_{\mathrm{di}}$ and $\alpha_{\mathrm{f}} = \alpha_{\mathrm{df}}$ as defined in Lemma 10. If the parameters are well-adjusted, for any s_σ, one can determine α_{i} and α_{f} by finding the second and third roots of $s_0 = s_\sigma - \sigma \cdot (P - H)$.

We now prove a lemma for the dependency of $H_{\mathrm{fmax},\sigma}$ as σ increases:

Lemma 11. *Let $\varepsilon \in \mathbb{R}_{>0}$. If s_{σ_1} and $s_{\sigma_1+\varepsilon}$ are well-shaped, one has $H_{fmax,\sigma_1+\varepsilon} < H_{fmax,\sigma_1}$.*

Proof. Let $\sigma_2 = \sigma_1 + \varepsilon$. Let $h = \kappa \frac{H^n}{H^n+K^n}(P - H)$. From Lemma 3 statement 2, for any H in the set $[0, +\infty[\setminus]H_{\mathrm{fmin},\sigma_1}, H_{\mathrm{fmax},\sigma_1}[$, one has $\frac{d}{dH} s_{\sigma_1}(H) \leq 0$. Thus, since $\frac{dh}{dH} = \sigma_1 + \rho + \frac{ds_{\sigma_1}}{dH}$ (see Equation (3)), for any H_{in} in $]H_{\mathrm{fmin},\sigma_1}, H_{\mathrm{fmax},\sigma_1}[$, and for any H_{out} in $[0, H_{\mathrm{fmin},\sigma_1}] \cup [H_{\mathrm{fmax},\sigma_1}, +\infty[$, one has $\frac{d}{dH} h(H_{\mathrm{in}}) > \frac{d}{dH} h(H_{\mathrm{out}})$. Because $H_{\mathrm{fmax},\sigma_1}$ is a local optimum of s_{σ_1} and $\frac{ds_{\sigma_1}}{dH}$ is continuous for H in $\mathbb{R}_+$, one has $\frac{d}{dH} h(H_{\mathrm{fmax},\sigma_1}) = \sigma_1 + \rho$. As $\sigma_2 > \sigma_1$, one has $\sigma_2 + \rho > \sigma_1 + \rho$. Since s_{σ_2} is well shaped, $H_{\mathrm{fmax},\sigma_2}$ exists. Because $H_{\mathrm{fmax},\sigma_2}$ is a local optimum of s_{σ_2} and $\frac{ds_{\sigma_2}}{dH}$ is continuous for H in $\mathbb{R}_+$, one has $\frac{d}{dH} h(H_{\mathrm{fmax},\sigma_2}) = \sigma_2 + \rho > \frac{d}{dH} h(H_{\mathrm{fmax},\sigma_1})$. Thus, the only possible value for $H_{\mathrm{fmax},\sigma_2}$ such that $\frac{dh}{dH}(H_{\mathrm{fmax},\sigma_2}) = \sigma_2 + \rho$ must be in $]H_{\mathrm{fmin},\sigma_1}, H_{\mathrm{fmax},\sigma_1}[$. □

Now, we prove that if the parameters are well-adjusted, there exists a critical rate σ_c such that s_{σ_c} has two roots, for any $\sigma < \sigma_c$, the function s_σ has one root, and for any $\sigma > \sigma_c$, the function s_σ has three roots. Thus, allowing us to prove the σ interval described in Theorem 1.

Lemma 12. *If the parameters are well-adjusted, there exists a σ_c such that s_{σ_c} only has two roots, and one of those roots is H_{fmin,σ_c}. Furthermore, if $0 \leq \sigma < \sigma_c$, s_σ has three roots. And, if $\sigma > \sigma_c$, s_σ has one root.*

Proof. For all σ in $\mathbb{R}_+$, s_σ is continuous for H in $\mathbb{R}_+$.

As the parameters are well-adjusted, Lemma 10 implies that the function $s_\sigma - \sigma \cdot (P - H) = s_0$ has three roots. Thus, Lemma 6 implies that s_0 is well-shaped and both $H_{\mathrm{fmin},0}$ and $H_{\mathrm{fmax},0}$ exist. From Lemma 3, $s_0(H)$ is decreasing for H within $[0, H_{\mathrm{fmin},0}]$ and increasing for H within $[H_{\mathrm{fmin},0}, H_{\mathrm{fmax},0}]$. Thus, one has $\arg\min_{H \in [0, H_{\mathrm{fmax},0}]} s_0(H) = H_{\mathrm{fmin},0}$ and, since s_0 has three roots, Lemma 6 implies $s_0(H_{\mathrm{fmin},0}) < 0$. Since $s_\sigma(H)$ is continuous for σ in $\mathbb{R}_+$, there exists an interval $\Omega = [0, \sigma_{\mathrm{dci}}[$, for which, for σ in Ω, one has $\min_{H \in [0, H_{\mathrm{fmax},0}]} s_\sigma(H) < 0$. Let σ_{dc} be the smallest σ_{dci} such that

$$\min_{H \in [0, H_{\mathrm{fmax},0}]} s_{\sigma_{\mathrm{dc}}}(H) = 0 \ . \tag{20}$$

One has that σ_{dc} is well-defined because: For any compact set interval $I \subset [0, P[$, let $H_{\mathrm{dmi}} = \arg\min_{H \in I} s_{\sigma_{\mathrm{dh}}}$. One has that H_{dmi} is well-defined as I is a compact set and s_σ is continuous for H in I. One can verify that there exists σ_{dh} such that for all H in I, one has $s_{\sigma_{\mathrm{dh}}}(H) > 0$, through the following inequality:

$$\sigma_{\mathrm{dh}} > \frac{\kappa \frac{H_{\mathrm{dmi}}^n}{K^n + H_{\mathrm{dmi}}^n}(P - H_{\mathrm{dmi}}) - \rho H_{\mathrm{dmi}}}{(P - H_{\mathrm{dmi}})} \tag{21}$$

Thus, the intermediate value theorem assures that σ_{dc} is finite and well-defined.

Let $H_{\mathrm{msc}} = \arg\min_{H \in [0, H_{\mathrm{fmax},0}]} s_{\sigma_{dc}}(H)$. One has that H_{msc} is well-defined as s_σ is continuous for H in $[0, H_{\mathrm{fmax},0}]$.

As $\sigma_{dc} > 0$ and Lemma 6 implies that $s_0(H_{\mathrm{fmax},0}) > 0$, Corollary 5 assures that:

$$s_{\sigma_{dc}}(H_{\mathrm{fmax},0}) > s_0(H_{\mathrm{fmax},0}) > 0 \tag{22}$$

From the definition of s_{σ_c}, one has

$$\lim_{H \to \infty} s_{\sigma_{\mathrm{dc}}}(H) = -\infty \ . \tag{23}$$

From Equations (22) and (23) and the intermediate value theorem,

$$\text{there is at least one root for } s_{\sigma_{\mathrm{dc}}} \text{ in }]H_{\mathrm{fmax},0}, +\infty[\ . \tag{24}$$

It follows from (20) and (24) that $s_{\sigma_{\mathrm{dc}}}$ has at least two roots, so Lemma 6 implies that $s_{\sigma_{dc}}$ is well-shaped.

If $s_{\sigma_{dc}}$ is well-shaped, Lemma 3 statement 2 implies that $s_{\sigma_{dc}}$ is decreasing within $[0, H_{\mathrm{fmin},\sigma_{dc}}]$ and increasing within the interval $[H_{\mathrm{fmin},\sigma_c}, H_{\mathrm{fmax},\sigma_{dc}}]$. Thus, s_{σ_c} has a unique minimum $H_{\mathrm{fmim},\sigma_{dc}}$ in $[0, H_{\mathrm{fmax},\sigma_{dc}}]$. One can then use equation (20) to deduce that s_σ has only one root in $[0, H_{\mathrm{fmax},\sigma_{dc}}]$ and it must be $H_{\mathrm{fmin},\sigma_{dc}}$.

From Lemma 3, one has that $s_{\sigma_{dc}}$ is strictly decreasing within $[H_{\mathrm{fmax},\sigma_{dc}}, +\infty[$. Therefore, for any H_1 and H_2, with $H_1 < H_2$ in $[H_{\mathrm{fmax},\sigma_{dc}}, +\infty[$, if $s_{\sigma_{dc}}(H_1) > 0$ and $s_{\sigma_{dc}}(H_2) > 0$, $s_{\sigma_{dc}}$ has no roots in $[H_1, H_2]$. Lemma 11 implies that

$$H_{\mathrm{fmax},\sigma_{dc}} < H_{fmax,0} \ . \tag{25}$$

From Equation (22), one has $s_{\sigma_{dc}}(H_{\mathrm{fmax},0}) > 0$. Thus, $s_{\sigma_{dc}}$ has no roots in $[H_{\mathrm{fmax},\sigma_{dc}}, H_{\mathrm{fmax},0}]$. It follows that $H_{\mathrm{fmim},\sigma_{dc}}$ is the only root $s_{\sigma_{dc}}$ has in $[0, H_{\mathrm{fmax},0}]$.

From Equation (25) and Lemma 3 implication that $s_{\sigma_{dc}}$ is strictly decreasing in $[H_{\mathrm{fmax},\sigma_0}, +\infty[$, one has that $s_{\sigma_{\mathrm{dc}}}$ is strictly decreasing in $[H_{\mathrm{fmax},\sigma_0}, +\infty[\subset [H_{fmax,\sigma_{dc}}, +\infty[$. Therefore, using Equation (24), one can conclude that $s_{\sigma_{\mathrm{dc}}}$ has only one root in $[H_{\mathrm{fmax},0}, +\infty[$.

One can conclude that $s_{\sigma_{dc}}$ has only two roots. The root $H_{fmin,\sigma_{dc}}$ in $[0, H_{fmax,0}]$ and the root $H_{\mathrm{feq},\sigma_{dc}}$ in $[H_{fmax,0}, +\infty[$.

From the definition of σ_{dc}, for any $\sigma < \sigma_{dc}$, one has $\min_{H \in [0, H_{\mathrm{fmax},0}]} s_0(H) < 0$. Therefore, as s_0 has three roots, Lemma 8 implies that for any $\sigma < \sigma_{\mathrm{dc}}$, the function s_σ has three roots.

Choose any $\sigma > \sigma_{\mathrm{dc}}$. We distinguish between s_σ being well-shaped or not:

- If s_σ is well-shaped for σ, Lemma 11 implies that $H_{fmax,\sigma} < H_{fmax,\sigma_{\mathrm{dc}}}$. Lemma 7 implies that for any H in $[0, H_{\mathrm{feq},\sigma_{dc}}]$, one has

$$s_\sigma(H) > s_{\sigma_{dc}}(H) \geq 0 \ . \tag{26}$$

Lemma 6 implies $H_{\text{feq},\sigma_{dc}} > H_{\text{fmax},\sigma_{dc}} > H_{\text{fmax},\sigma}$. Since Lemma 3 implies that s_σ is strictly decreasing for values larger than $H_{\text{fmax},\sigma}$, the function s_σ can have at most one root greater than

$$H_{\text{fmax},\sigma} < H_{\text{fmax},0} \ . \tag{27}$$

From Lemma 6, stating that s_σ has between one and three roots, and Equations (26) and (27), the function s_σ must have exactly one root in $]H_{\text{feq},\sigma_{\text{dc}}}, +\infty[$.

- If s_σ is not well-shaped for σ, then Lemma 6 guarantees it will only have one root.

The lemma's statement follows from setting $\sigma_{\text{c}} = \sigma_{\text{dc}}$. □

We will use the notation of σ_{c} as defined in Lemma 12 in the following lemmas. We now use the Lyapunov stability theorem to prove convergence of $H(t)$ to a root as t approaches $+\infty$.

Lemma 13. *If the parameters are well-adjusted and σ in $[0, \sigma_c[$:*

- *If $H(0)$ is in $[0, H_{ceq,\sigma}[$, then $H(t)$ converges to $H_{ieq,\sigma}$.*
- *If $H(0)$ is in $]H_{ceq,\sigma}, +\infty[$, then $H(t)$ converges to $H_{feq,\sigma}$.*

Proof. Let $H(0)$ in $[0, H_{\text{ceq},\sigma}[$. If the parameters are well adjusted and σ in $[0, \sigma_c[$, Lemma 12 assures that s_σ has three roots. Thus, one has that the three roots $H_{\text{ieq},\sigma} < H_{\text{ceq},\sigma} < H_{\text{feq},\sigma}$ exist. We choose the Lyapunov function V_1 from $\mathbb{R}$ to $\mathbb{R}$ as:

$$V_1(H) = (H_{\text{ieq},\sigma} - H)^2$$
$$\frac{d}{dH}V_1(H) = -2(H_{\text{ieq},\sigma} - H)$$

Let $\Omega = [0, H_{\text{ceq},\sigma}[$. If both statements hold: *(1)* $s_\sigma(H)\frac{d}{dH}V_1(H) < 0$ for all H in Ω except for a root of s_σ denoted by H_r that is in Ω and *(2)* $H(0)$ is in Ω. The Lyapunov stability condition guarantees $H(t)$ will converge to the root H_r inside Ω.

From Corollary 4, one has that s_σ is positive within $[0, H_{\text{ieq},\sigma}[$. From the definition of V_1, it is $\frac{dV_1}{dH}(H) < 0$ for H in $[0, H_{\text{ieq},\sigma}[$. Thus, one has $s_\sigma(H)\frac{d}{dH}V_1(H) < 0$ for H in $[0, H_{\text{ieq},\sigma}[$. From Corollary 4, one has that s_σ is negative within $]H_{\text{ieq},\sigma}, H_{\text{ceq},\sigma}[$. From the definition of V, $\frac{d}{dH}V_1(H) > 0$ for H in $]H_{\text{ieq},\sigma}, H_{\text{ceq},\sigma}[$. Thus, one has $s_\sigma(H)\frac{d}{dH}V_1(H) < 0$ for H in $]H_{\text{ieq},\sigma}, H_{\text{ceq},\sigma}[$. It follows that $s_\sigma(H)\frac{d}{dH}V_1(H) < 0$ for H in $[0, H_{\text{ceq},\sigma}[$ excluding $H_{\text{ieq},\sigma}$, so the Lyapunov stability condition assures $H(t)$ converges to $H_{\text{ieq},\sigma}$.

One can repeat the same argument for the root $H_{\text{feq},\sigma}$ using the Lyapunov function $V_2(H) = (H_{\text{feq},\sigma} - H)^2$, to conclude $H(t)$ converges to $H_{\text{feq},\sigma}$ if $H(0)$ in $]H_{\text{ceq},\sigma}, +\infty[$. □

Lemma 14. *If the parameters are well-adjusted and σ in $]\sigma_c, +\infty[$: If $H(0)$ in $[0, +\infty[$, then $H(t)$ converges to $H_{feq,\sigma}$*

Proof. From Lemma 12, the only root of s_σ is $H_{\text{feq},\sigma}$. The function s_σ is continuous for H in $\mathbb{R}_+$. One has that $s_\sigma(0) > 0$, and also that $\lim_{H\to+\infty} s_\sigma = -\infty$. From the previous statements, it follows that $s_\sigma(H) > 0$ for H in $[0, H_{\text{feq},\sigma}[$ and $s_\sigma(H) < 0$ for H in $]H_{\text{feq},\sigma}, +\infty[$.

By using the following Lyapunov function V for the interval $[0, +\infty[$

$$\begin{aligned} V(H) &= (H_{\text{feq},\sigma} - H)^2 \\ \frac{d}{dH}V(H) &= -2(H_{\text{feq},\sigma} - H) \end{aligned} \tag{28}$$

one has $s_\sigma(H)\frac{d}{dH}V(H) < 0$ for H in $[0, +\infty[$ except for $H = H_{feq,\sigma}$. Thus, $H(t)$ converges to $H_{\text{feq},\sigma}$. □

The following lemma is similar to Lemma 8, but it removes the constraint requiring s_σ to have three roots and it applies only to $H_{\text{feq},\sigma}$.

Lemma 15. *Let $\varepsilon > \mathbb{R}_+$. One has $H_{feq,\sigma+\epsilon} > H_{feq,\sigma}$.*

Proof. As $s_{\sigma+\varepsilon}$ is continuous for H in $\mathbb{R}_+$, the intermediate value theorem applies. From Corollary 5, one has that $s_{\sigma+\varepsilon}(H_{\text{feq},\sigma}) > s_\sigma(H_{\text{feq},\sigma}) = 0$ (29). From the definition of $s_{\sigma+\varepsilon}$, one has the following: $\lim_{H\to+\infty} s_{\sigma+\varepsilon} = -\infty$ (30). From Equations (29) and (30), and the intermediary value theorem there must be at least one root in $]H_{\text{feq},\sigma}, +\infty[$. Thus, $H_{\text{feq},\sigma+\epsilon} > H_{\text{feq},\sigma}$. □

Towards the goal of proving the converge to the set $[\alpha_f P, P[$ in Theorem 1, we establish a result on the position of $H_{\text{feq},\sigma}$.

Lemma 16. *Let the parameters be well-adjusted. Let α_i and α_f be as defined in Lemma 10. Then $H_{feq,\sigma} \in [\alpha_f P, P[$. If $\sigma \in [0, \sigma_c[$, $H_{ieq,\sigma} \in [0, \alpha_i P[$. Thus, the distance between $H_{ieq,\sigma}$ and $H_{feq,\sigma}$ is at least $(\alpha_f - \alpha_i)P$.*

Proof. As the parameters are well-adjusted Lemma 10 implies that $s_\sigma - \sigma(P-H) = s_0$ has three roots, so $\alpha_i = \frac{H_{\text{ceq},0}}{P}$ and $\alpha_f = \frac{H_{\text{feq},0}}{P}$ are well-defined. From Lemma 15, one has $H_{\text{feq},\sigma} > H_{\text{feq},0}$. Thus, Lemma 4 implies $H_{\text{feq},\sigma} \in [\alpha_{\text{f}} P, P[$.

If $\sigma \in [0, \sigma_{\text{c}}[$, from Lemma 8 and Lemma 6, $H_{\text{ieq},\sigma} < H_{\text{ceq},\sigma} < H_{\text{ceq},0}$. Thus, $H_{\text{ieq},\sigma} \in [0, \alpha_{\text{i}} P[$.

It follows that $H_{\text{feq},\sigma} - H_{\text{ieq},\sigma} > H_{\text{feq},0} - H_{\text{ceq},0} > (\alpha_2 - \alpha_1)P$; from which the lemma's statement follows. □

Combining Lemmas 13, 14, 15, 16, as well as setting $H_{\text{c},\sigma} = H_{\text{ceq},\sigma}$, the statement of Theorem 1 follows.

Proof of Theorem 2

We start by defining a lower bound function b_σ for s_σ. We use the index σ to emphasize that the lower bound function b_σ depends on σ. If the parameters are well-adjusted, we define $M_\sigma =$

$\min_{H\in[0,H_{\mathrm{ceq},0}]} s_\sigma(H)$. We further define the function b_σ from $\mathbb{R}_+$ to $\mathbb{R}$ as:

$$b_\sigma(H) = M_\sigma - \frac{M_\sigma}{H_{\mathrm{feq},\sigma}} H \tag{31}$$

As s_σ is continuous, the set $[0, H_{\mathrm{ceq}}, 0]$ is a compact set, and Lemma 6 guarantees that $H_{\mathrm{feq}} > 0$ exists, one has that M_σ and b_σ are well-defined.

We now prove that b_σ(H) is indeed a lower bound for s_σ(H) for H in $[0, H_{\mathrm{ceq},0}]$.

Lemma 17. *Let the parameters be well-adjusted. If σ in $]\sigma_c, +\infty[$, for any H_1 and H_2 in $[0, H_{ceq,0}]$, one has $s_\sigma(H_1) \geq b_\sigma(H_2) > 0$.*

Proof. As the parameters are well-adjusted, Lemma 10 implies that $s_0 = s_\sigma - \sigma\cdot(P-H)$ has three roots. Thus, one has that $H_{\mathrm{ieq},0}$, $H_{\mathrm{ceq},0}$, and $H_{\mathrm{feq},0}$ exist, as well as $[0, H_{\mathrm{ceq},0}] \subset [0, H_{\mathrm{feq},0}]$. From Lemma 15, one has that $[0, H_{\mathrm{feq},0}] \subset [0, H_{\mathrm{feq},\sigma}[$. So,

$$H_{\mathrm{ceq},0} < H_{\mathrm{feq},\sigma} \tag{32}$$

and

$$[0, H_{\mathrm{ceq},0}] \subset [0, H_{\mathrm{feq},\sigma}[\ . \tag{33}$$

Further, s_σ is continuous for H in $\mathbb{R}_+$. Since σ in $]\sigma_{\mathrm{c}}, \infty[$, Lemma 12 guarantees that s_σ has only one root. From the definition of s_σ, it is $s_\sigma(0) = \sigma P > 0$, and $\lim_{H\to+\infty} s_\sigma = -\infty$. From the three latter statements, one has

$$s_\sigma(H) > 0 \text{ for } H \text{ in } [0, H_{\mathrm{feq},\sigma}[\ . \tag{34}$$

From statements (33) and (34), it follows that $s_\sigma(H) > 0$ for H in $[0, H_{\mathrm{ceq},0}]$.

One has that $b_\sigma(0) = M_\sigma = \min_{H\in[0,H_{\mathrm{ceq},0}]} s_\sigma(H) \leq s_\sigma(H)$ for all H in $[0, H_{\mathrm{ceq},0}]$. From the definition of b_σ, this function is strictly decreasing for H in $\mathbb{R}$. It follows that the inequality $s_\sigma(H_1) \geq b_\sigma(H_2)$ holds for all H_1 and H_2 in $[0, H_{\mathrm{ceq},0}]$.

From Equation (32), $H_{\mathrm{ceq},0} < H_{\mathrm{feq},\sigma}$. From Equation (34), one has the following inequality: $b_\sigma(0) = M_\sigma = \min_{H\in[0,H_{\mathrm{ceq},0}]} s_\sigma(H) \leq 0$. By definition, b_σ is an affine function positive for any $H < H_{\mathrm{feq},\sigma}$ and negative for $H > H_{\mathrm{feq},\sigma}$. It follows that $b_\sigma(H)$ is positive for H in $[0, H_{\mathrm{ceq},0}]$. □

The next result is folklore for ODEs. We provide a proof for the sake of completeness.

Lemma 18. *Let $\frac{dx}{dt} = f(x)$, where f is a function from $\mathbb{R}$ to $\mathbb{R}$. A solution $x(t)$ of this ODE is monotonic.*

Proof. Let us assume by contradiction that $x(t)$ is non-monotonic. This implies that there exist times t_1 in $\mathbb{R}^+$, t_2 in $\mathbb{R}^+$, and t_{m} in $\mathbb{R}^+$ with $t_1 < t_{\mathrm{m}} < t_2$ such that $x(t_1) = x(t_2)$ and $x(t_{\mathrm{m}}) \neq x(t_2)$. Consider the integral from time t_1 to t_2:

$$\int_{t_2}^{t_2} f(x)\frac{dx}{dt}dt \tag{35}$$

By integrating through substitution, one has:

$$\int_{x(t_1)}^{x(t_2)} f(z)dz = 0 \tag{36}$$

Which is equal to zero as $x(t_2) = x(t_1)$. However, since $\frac{dx}{dt} = f(x)$, the first integral also yields:

$$\int_{t_2}^{t_2} (f(x))^2 dt = 0 \tag{37}$$

Since $(f(x))^2 \geq 0$ and $f(x)$ has only asymptotic discontinuities if it is not continuous, the value of the integral is only zero if $f(x)$ is zero almost everywhere. This implies that $x(t)$ is constant for t in $[t_1, t_2]$. It follows that $x(t_\mathrm{m}) = x(t_1) = x(t_2)$, which is a contradiction to the assumption that $x(t)$ is non-monotonic. □

Lemma 19. *Let the parameters be well adjusted. Let $H_{ceq,0}$ be the second largest root of s_0. Let σ be in $]\sigma_c, +\infty[$ and assume that for the initial density $H(0) = 0$.*

Then, there exists a time t_h such that for any $t > t_h$, one has $H(t) \in]H_{ceq,0}, +\infty[$. Further, $t_h \leq -\ln\left(1 - \frac{H_{ceq,0}}{H_{feq,\sigma}}\right) \cdot \frac{H_{feq,\sigma}}{M_\sigma}$.

Proof. Consider the two ODEs $\frac{dH^*}{dt} = b_\sigma(H^*) = M_\sigma - \frac{M_\sigma}{H_{\mathrm{feq},\sigma}} H^*$ with $H^*(0) = 0$ and $\frac{dH}{dt} = s_\sigma(H)$ with $H(0) = 0$.

From Lemma 17, one has that $s_\sigma = \frac{dH}{dt} > 0$ for H in $[0, H_{\mathrm{ceq},0}]$. Thus, Lemma 18 implies the function $H(t)$ is strictly increasing while $H(t)$ is in $[0, H_{\mathrm{ceq},0}]$. It follows that there exists a finite time interval $I_1 = [0, t_{\mathrm{dh}}]$ such that for any t in I, one has $H(t) \in [0, H_{\mathrm{ceq},0}]$ and for any t not in I, one has $H(t) \in]H_{\mathrm{ceq},0}, +\infty[$. We will later show that we may set $t_\mathrm{h} = t_{\mathrm{dh}}$.

From Lemma 17, one has that $b_\sigma(H^*) = \frac{dH^*}{dt} > 0$ for H^* in $[0, H_{\mathrm{ceq},0}]$. Thus, Lemma 18 implies the function $H^*(t)$ is strictly monotonic increasing while $H^*(t)$ is in $[0, H_{\mathrm{ceq},0}]$. It follows that there exists a there must exist a finite time interval $I_2 = [0, t_{\mathrm{db}}]$ for which for any t in I, one has $H^*(t) \in [0, H_{\mathrm{ceq},0}]$ and for any t not in I, one has $H^*(t) \in]H_{\mathrm{ceq},0}, +\infty[$.

Lemma 17 guarantees that for any H and H^* in $[0, H_{\mathrm{ceq},0}]$, one has $s_\sigma(H) \geq b_\sigma(H^*)$. Thus, for any t in the intersection $I_{\mathrm{int}} = [0, t_{\mathrm{dh}}] \cap [0, t_{\mathrm{db}}]$, one has that $H(t)$ and $H^*(t)$ are both in $[0, H_{\mathrm{ceq},0}]$. As $H(0) = H^*(0) = 0$, it is for any t in I_{int}:

$$\int_0^t s_\sigma(H(\tau))d\tau \geq \int_0^t b_\sigma(H^*(\tau))d\tau \tag{38}$$

As b_σ and s_σ are continuous for H and H^* in $\mathbb{R}_+$, they are integrable in any compact set, thus both integrals are well-defined. From the definition of t_{dh} and t_{db}, it is:

$$H_{ceq,\sigma} = \int_0^{t_{\mathrm{dh}}} s_\sigma(H(\tau))d\tau$$
$$H_{ceq,\sigma} = \int_0^{t_{\mathrm{db}}} b_\sigma(H^*(\tau))d\tau$$

Therefore:

$$\int_0^{t_{\mathrm{dh}}} s_\sigma(H(\tau))d\tau = \int_0^{t_{\mathrm{db}}} b_\sigma(H^*(\tau))d\tau \tag{39}$$

By means of contradiction, let us assume that $t_{\mathrm{dh}} > t_{\mathrm{db}}$. Thus, $[0, t_{\mathrm{db}}] \subset [0, t_{\mathrm{dh}}]$ and $I_{int} = [0, t_{\mathrm{dh}}]$. Lemma 17 implies $s_\sigma(H) > 0$ for H in $[0, H_{\mathrm{ceq},0}]$. Thus, for t in $[0, t_{\mathrm{dh}}]$, one has $H(t)$ in $[0, H_{\mathrm{ceq},0}]$ with $s_\sigma(H(t)) > 0$ and $s_\sigma(H(t))$ is strictly positive in the interval I_{int} from (39). It follows from the assumption $t_{\mathrm{db}} < t_{\mathrm{dh}}$:

$$\int_0^{t_{\mathrm{db}}} s_\sigma(H(\tau))d\tau < \int_0^{t_{\mathrm{db}}} b_\sigma(H^*(\tau))d\tau \tag{40}$$

Equation (40) contradicts Equation (38). Thus one can conclude that $t_{\mathrm{dh}} \leq t_{\mathrm{db}}$; showing that the lemma's first statement holds for $t_{\mathrm{h}} = t_{\mathrm{dh}}$.

Finally, the ODE $\frac{dH^*}{dt} = M_\sigma - \frac{M_\sigma}{H_{\mathrm{feq},\sigma}} H^* = b_\sigma(H^*)$ with $H^*(0) = 0$ has as unique solution:

$$H^*(t) = H_{\mathrm{feq},\sigma}\left(1 - e^{-\frac{M_\sigma \cdot t}{H_{\mathrm{feq},\sigma}}}\right)$$

Solving for t_{db} yields $t_{\mathrm{db}} = -\frac{H_{\mathrm{feq},\sigma}}{M_\sigma} \cdot \ln\left(1 - \frac{H_{\mathrm{ceq},0}}{H_{\mathrm{feq},\sigma}}\right)$; from which the lemma's second statement follows.

□

With Lemma 19, we provide a minimum time necessary for $H(t)$ to leave the interval $[0, H_{\mathrm{ceq},0}]$. We now prove that if $H(t)$ has left this interval, it will converge to $[\alpha_{\mathrm{f}} P, P[$.

Lemma 20. *Let the parameters be well-adjusted. If there exists a time t_{in} such that $H(t_{in})$ is in $[H_{ceq,0}, +\infty[$, then $H(t)$ converges to $H_{feq,\sigma}$ in $[\alpha_f P, P[$.*

Proof. Recall that $\frac{dH}{dt} = s_\sigma$. Since s_σ does not depend on time t, we may apply a time shift and solve the ODE $\frac{dH^s}{dt_s} = s_\sigma$ where $t_s = t - t_{\mathrm{in}}$ with $t_s \geq t_{\mathrm{in}}$ and $H^s(0) = H(t_{\mathrm{in}})$. Further, for all t in $[t_{\mathrm{in}}, +\infty[$, one has $H^s(t - t_{\mathrm{in}}) = H(t)$. Thus, $H^s(t - t_{\mathrm{in}})$ and $H(t)$ converge to the same value as t goes to infinity. One can now apply Theorem 1 using $H(t_{\mathrm{in}})$ as the initial value $H^s(0) = H(t_{\mathrm{in}})$ to obtain convergence. We next distinguish between different cases for σ:

Case σ in $[0, \sigma_c[$: Lemma 12 implies that s_σ has three roots. Lemma 8 implies that $H_{ceq,\sigma} < H_{ceq,0} < H^s(0) = H(t_{\mathrm{in}})$, thus Theorem 1 implies that $H^s(t)$ converges to $H^s_{\mathrm{feq},\sigma}$ in $[\alpha_f P, P[$.

Case σ in $]\sigma_c, +\infty[$: As $[H_{\mathrm{ceq},0}, +\infty[\subset [0, +\infty[$, Theorem 1 implies that $H^s(t)$ converges to $H_{\mathrm{feq},\sigma}$ in $[\alpha_f P, P[$.

Case $\sigma = \sigma_c$: Lemma 10 implies that s_{σ_c} has two roots, so Lemma 6 implies that s_{σ_c} is well-shaped. Thus, one can apply Lemma 11 to conclude $H_{\mathrm{fmax},\sigma_c} < H_{\mathrm{fmax},0}$. Corollaries 4 and 5 imply that $s_{\sigma_c}(H) > s_0(H) \geq 0$ for any H in $[H_{\mathrm{ceq},0}, H_{\mathrm{fmax},0}]$. Since Lemma 3 implies that s_{σ_c} is strictly decreasing for $H^s > H_{\mathrm{fmax},\sigma_c}$ and $\lim_{H \to +\infty} s_{\sigma_c}(H) = -\infty$, the function s_{σ_c} has only

one root in $[H_{\text{fmax},0}, +\infty[\subset [H_{\text{fmax},\sigma_c}, +\infty[$, which must be H_{feq,σ_c}. Since H_{feq,σ_c} is contained in $[H_{\text{fmax},0}, +\infty[$ and $H_{\text{ceq},0} < H_{\text{fmax},0}$, it is

$$H_{\text{ceq},0} < H_{\text{feq},\sigma_c} \tag{41}$$

It follows that $s_{\sigma_c}(H) > 0$ for H in $[H_{\text{ceq},0}, H_{\text{feq},\sigma_c}[$ and $s_{\sigma_c}(H) < 0$ for H in $[H_{\text{feq},\sigma_c}, +\infty[$.

From Equation (41), the Lyapunov function $V(H) = (H_{\text{feq},\sigma_c} - H)^2$ for H in $[H_{\text{ceq},0}, +\infty[$ fulfills $\frac{d}{dH}V(H) < 0$ for H in $[H_{\text{ceq},0}, H_{\text{feq},\sigma_c}[$ and $\frac{d}{dH}V(H) > 0$ for H in $[H_{\text{feq},\sigma_c}, +\infty[$.

It follows from the product $s_{\sigma_c} \cdot \frac{d}{dH}V(H)$ that $H^s(t)$ converges to H_{feq,σ_c}.

Finally, Lemma 16 guarantees that H_{feq,σ_c} is in $[\alpha_{\text{f}}P, P[$; which concludes the proof. □

With Lemma 20, one observes that the Allee-based algorithm needs to be exposed to a rare event rate for a time at least $\frac{H_{\text{feq},\sigma}}{M_\sigma} \cdot \ln\left(1 - \frac{H_{\text{ceq},0}}{H_{\text{feq},\sigma}}\right)$ to guarantee that the hold functionality triggers. Once $H(t) > H_{\text{ceq},0}$, the H cell density $H(t)$ will converge to $[\alpha_{\text{f}}P, P[$, no matter the value of σ after this time.
